# Can Tai Chi and Qigong Postures Shape Our Mood? Toward an Embodied Cognition Framework for Mind-Body Research

**DOI:** 10.3389/fnhum.2018.00174

**Published:** 2018-05-01

**Authors:** Kamila Osypiuk, Evan Thompson, Peter M. Wayne

**Affiliations:** ^1^Osher Center for Integrative Medicine, Division of Preventive Medicine, Brigham and Women's Hospital, Harvard Medical School, Boston, MA, United States; ^2^Department of Philosophy, University of British Columbia, Vancouver, BC, Canada

**Keywords:** posture, Tai Chi, Qigong, embodied cognitive science, mood, depression, embodiment

## Abstract

Dynamic and static body postures are a defining characteristic of mind-body practices such as Tai Chi and Qigong (TCQ). A growing body of evidence supports the hypothesis that TCQ may be beneficial for psychological health, including management and prevention of depression and anxiety. Although a variety of causal factors have been identified as potential mediators of such health benefits, physical posture, despite its visible prominence, has been largely overlooked. We hypothesize that body posture while standing and/or moving may be a key therapeutic element mediating the influence of TCQ on psychological health. In the present paper, we summarize existing experimental and observational evidence that suggests a bi-directional relationship between body posture and mental states. Drawing from embodied cognitive science, we provide a theoretical framework for further investigation into this interrelationship. We discuss the challenges involved in such an investigation and propose suggestions for future studies. Despite theoretical and practical challenges, we propose that the role of posture in mind-body exercises such as TCQ should be considered in future research.

## Introduction

Tai Chi and Qigong (TCQ) are two increasingly popular mind-body interventions being therapeutically used and medically prescribed for the prevention and rehabilitation of a wide range of health conditions, including mood disorders (Lauche et al., 2016). TCQ share a common history that includes elements of traditional Chinese medicine, martial arts conditioning, and Asian lifestyle philosophy (Wayne and Fuerst, 2013). Both are inherently multi-modal, and typically integrate flowing movements, dynamic and static postural training, and breath instruction, along with training in a variety of cognitive skills including heightened somatic awareness, imagery, and focused mental attention (Wayne and Kaptchuk, 2008a,b; Payne and Crane-Godreau, 2013; Wayne et al., 2013). Both are considered forms of “Meditative Movement” (Larkey et al., 2009). For these reasons, in the present work TCQ are grouped together and considered equivalent interventions, following other recent reviews (Jahnke et al., 2010; Payne and Crane-Godreau, 2013; Klein et al., 2016).

A growing body of evidence indicates that TCQ may be helpful in the treatment and management of depression, anxiety, and related mood disorders (Jahnke et al., 2010; Wang et al., 2010a, 2014; Chi et al., 2013; Payne and Crane-Godreau, 2013; Liu et al., 2015). This evidence adds to research showing positive benefits of conventional exercise on psychological well-being (Asmundson et al., 2013; Jayakody et al., 2014; Catalan-Matamoros et al., 2016; Kvam et al., 2016; Stubbs et al., 2017). Although few large scale randomized trials have been conducted to evaluate TCQ specifically for primary complaints of mood disorders, multiple smaller trials (Chou et al., 2004; Liu et al., 2015; Caldwell et al., 2016; Yeung et al., 2017) and larger trials evaluating depression and anxiety comorbid with other conditions (e.g., pain, heart failure, cancer) (Wang et al., 2010b, 2016; Yeh et al., 2013; Tao et al., 2016; Wieczorrek et al., 2016) collectively suggest that TCQ may be an effective and safe non-pharmacological therapy for preventing and managing mood disorders [see Payne and Crane-Godreau (2013) and Saeed et al. (2010) for comprehensive reviews].

Integral to research evaluating the effectiveness of TCQ and related mind-body therapies for psychological health is examination of the physiological and psychosocial factors underlying clinical changes. Plausible causal models add credibility and contribute to the totality of evidence for novel interventions. Knowledge of principal causal factors also informs efforts to improve and/or tailor interventions to suit the needs of specific populations. Because of the multimodal nature of TCQ, it is likely that observed clinical benefits result from multiple interacting causal factors (Wayne and Kaptchuk, 2008a,b; Payne and Crane-Godreau, 2013; Wayne et al., 2013; Klein et al., 2016). A number of physiological and psychosocial factors have been hypothesized to explain TCQ's therapeutic effects on psychological well-being. Some of these, such as moderate aerobic activity linked to increased levels of neurotrophic factors implicated in mood disorders (Hashimoto, 2010; Coelho et al., 2013; Matta Mello Portugal et al., 2013; Meyer et al., 2016; Castrén and Kojima, 2017), overlap with those attributed to conventional exercise. Others including breath and imagery-related changes in autonomic tone (Lang et al., 1980; Carroll et al., 1982; Bernardi et al., 2001; Wang S. Z. et al., 2010), heightened sensorimotor acuity (Jacobson et al., 1997; Kerr et al., 2008; Li and Manor, 2010; Manor et al., 2013; Chang et al., 2016; Cheng et al., 2017; Lauche et al., 2017; Wang et al., 2017), decreased catastrophizing and rumination (Hall et al., 2016), and enhanced psychosocial support (Taylor-Piliae et al., 2006; Tsang and Fung, 2008; Wayne and Kaptchuk, 2008b; Yang et al., 2011; Waite-Jones et al., 2013; Yeh et al., 2013; Fischer et al., 2014) may be unique to, or more greatly emphasized in, TCQ and related mind-body exercises. However, surprisingly absent from nearly all discussions of the casual factors contributing to the therapeutic effects of TCQ on mood and health in general is perhaps its most obvious characteristic—body posture and its dynamics (Payne and Crane-Godreau, 2013; Wayne and Fuerst, 2013; Schmalzl et al., 2014).

When observed from the outside, TCQ are characterized by a series of postures, sometimes dynamically linked together through detailed choreographed routines, and sometimes practiced as static postures or repeated simplified movement phrases. In both classical and modern training texts, great emphasis is placed on accurate performance of these postures along with the internal qualities that generate them. Examples of this instruction from classical texts include: “*The postures should be without defect, without hollows or projections from the proper alignment”; “Every joint in your body must be strung together. This allows Qi to pass smoothly through your body and benefits both form and application”; “Stand like a perfectly balanced scale and move like a turning wheel”; and “Keep the tailbone (coccyx) centrally aligned and straight so the spirit of vitality (shen) penetrates up to the crown of the head. Then, with the head feeling as if suspended from above, the entire body will be light and agile”* (Lo et al., 1979). The last of these quotations, in particular, highlights a core principle strived for in TCQ training, namely, a relaxed and apparently effortless quality employed in animating and supporting postures and their dynamics. This TCQ principle is referred to as “*Song*” (Lo et al., 1979). Sometimes over-simplistically translated as “relaxed,” *Song* is not a neuro-muscularly limp hypotonic state, but rather, a highly balanced state of tone. Payne and Crane-Godreau characterize *Song* as a quality that is “experienced as light, free, open and effortless; but at the same time, stable, powerful, and well-rooted” (Payne and Crane-Godreau, 2013). Importantly, they also emphasize this balanced state of postural tone is not solely due to “unconscious postural engineering rules or principles of biomechanical alignment,” but also is shaped by a rich “suite of dynamic, interoceptively rich, intentional qualities.” Thus, at the core of TCQ training is a dialectical (or ecological) co-creation of body postures and mental states, whereby physical shapes facilitate mental qualities and mental states inform physical shapes. Borrowing from Zen Buddhist teacher Shunryu Suzuki's first words on posture instruction for meditation in his classic book, *Zen Mind, Beginner's Mind*, “These forms are not the means of obtaining a right state of mind. To take this posture is itself to have the right state of mind” (Suzuki, 1970).

Although the interdependent relationship between body posture and mental state has not been experimentally evaluated within contemporary research on TCQ or other mind-body practices [e.g., yoga, meditation; but see Shapiro et al. (Shapiro and Cline, 2004)], the expanding field of embodied cognitive science provides a theoretical framework with supporting empirical evidence for investigating this relationship (Varela et al., 1991; Barrett, 2011). Embodied cognitive science emphasizes that the body not only plays a strong causal role in supporting cognitive processes but also that bodily processes can serve as proper parts of the cognitive processes themselves (Shapiro, 2010). For example, models of embodied cognition emphasize that higher level conceptual processes, as well as chronic mood states, are fundamentally grounded in bodily experiences (Gibbs, 2006; Handbook of Cognitive Science, 2008; Barsalou, 2010). Research focused on gestures has highlighted the coordination of talk with bodily action, demonstrating the multimodal nature of communication, including expression of emotion (Goldin-Meadow, 2003; McNeill, 2005). Research on visual perception has shown that self-generated bodily movement directly affects how and what we perceive (Noe, 2005; Wexler and van Boxtel, 2005). In general terms, one of the principal aims of embodied cognitive science is to devise explanatory models that specify how the body, brain, and environment mutually interact and make up a larger dynamical system in which the organism adaptively functions (Thompson, 2007; Chemero, 2011).

A primary goal of this paper is to draw from embodied cognitive science to propose a new perspective for investigating the relationship between bodily postures and mental states in TCQ. To this end, we call attention to and summarize already existing research evidence for the interdependent relationship between posture and psychological processes and its relevance to research evaluating the health benefits of mind-body therapies. We begin by providing an evolutionary framework for understanding the interdependence of posture and emotion. We call attention to the role of body language in the communication and perception of emotion and how it may have shaped the complex biology underlying links between posture and psychological states. We then provide examples from both experimental and observational research demonstrating how feedback from both the body and facial expressions influences emotion and cognition, and conversely, how affect influences physical posture and movement. We then consider theoretical and practical methodological challenges in evaluating the interdependence of posture and mood in the context of TCQ, and provide suggestions for future research.

### An evolutionary framework for understanding the interdependence of posture and emotion

Some of the earliest theories of emotion addressed the role of the body in the expression and experience of emotion. In *The Expression of the Emotions in Man and Animals*, Charles Darwin described in detail the observed physical expressions of emotion, noting that similar patterns of body movements are associated with specific states of mind. He understood these gestures to play an important role in communication, being readily perceived by others, giving “vividness and energy to” and “reveal[ing] the thoughts and intentions of others more truly than” spoken words (Darwin, 1872). Darwin identified a contracted, flaccid, downwardly sinking posture as being reflective of depression and grief, in contrast to the erect, upright, open posture of high spirits and cheerfulness. Darwin even touched upon the reciprocal nature of emotions and their physical expressions, stating, “The free expression by outward signs of an emotion intensifies it…These results follow partly from the intimate relation which exists between almost all the emotions and their outward manifestations; and partly from the direct influence of exertion on the heart, and consequently on the brain. Even the simulation of an emotion tends to arouse it in our minds” (Darwin, 1872).

Diverse psychological theories have acknowledged the role of the physical body in the expression, perception, and experience of emotions. William James proposed that the body is essential for the experience of emotion, which he viewed as the bodily experience of physiological changes resulting from the perception of an emotion-triggering stimulus (James, 1884). Proposed circuits of action of the body's role in emotion have included a reliance on a cognitive interpretation of the physical expression (Bem, 1972; Laird, 1974) or direct physiological feedback (Ekman et al., 1983; Strack et al., 1988). Daryl Bem's theory of self-perception suggests that people understand their emotional state through interpretation of their own expressive behavior (Bem, 1972). Antonio Damasio has acknowledged that emotion involves changes in both the body and the brain and that these changes are intertwined through complex feedback circuits (Damasio, 1995) (see also Colombetti and Thompson, 2007).

Contemporary empirical studies uphold the interdependency between the physical expressions of emotions and their role in social communication. Evidence indicates that emotions are not only displayed through facial expressions, but also through a dynamic whole-body language including both postures and movements (Atkinson et al., 2004; McHugh et al., 2010; Schneider et al., 2014). Positive emotions continue to be associated with an upright, open posture, and can be observed even in young children (Lewis et al., 1992). An expansive posture has been identified as the prototypical expression of pride and was found to be displayed by both sighted and blind athletes from across the globe in response to success, suggesting that such expressions are culturally universal and may have an evolutionary and biological foundation (Tracy and Matsumoto, 2008). In contrast, individuals depicted in a slumped posture have been perceived as holding depressed and helpless beliefs about themselves (Riskind and Gotay, 1982).

Even during the first year of life, infants are able to perceive emotion from both faces and from body movements (Zieber et al., 2014). Several studies have shown that observers are able to identify emotion from gait characteristics (Montepare et al., 1987; Schneider et al., 2014) and body postures (Coulson, 2004; Aviezer et al., 2012), with accuracy rates in some cases equal to (Coulson, 2004) or greater than (Aviezer et al., 2012) those of recognition from isolated facial expressions. Contemporary studies using fMRI provide evidence of a neural basis supporting recognition of emotion from the body. A specific area within the fusiform gyrus, a brain region associated with recognition of faces, has been identified as being selectively activated in response to images of human bodies, even when shown without faces (Peelen and Downing, 2005). Emotional body postures have also been shown to activate the amygdala (Hadjikhani and de Gelder, 2003). Some of Paul Ekman's work indicates that the information communicated by the body may be of a different nature than that communicated by facial expressions (Ekman, 1965). This work suggests a unique role of bodily expressions in the social communication of emotion.

The work of Ekman, Levenson, and Davidson has identified some of the specific neural and physiological changes that occur during various emotions (Ekman et al., 1983; Levenson et al., 1990; Ekman and Davidson, 1993; Levenson and Ekman, 2002). These changes have also been induced by voluntary manipulation of facial expressions through muscle contraction (Ekman et al., 1983; Ekman and Davidson, 1993). Given the communicative power of facial expressions, body postures, and movements associated with specific emotions, it is possible that the body and face not only communicate emotion externally to others, but also influence emotional and cognitive processes of the individual through feedback circuits mediated by neural and chemical signals (Damasio, 1995). Further evidence of the reciprocal relationship between emotional physical expressions and subjective feelings is explored in the sections below.

### Effects of emotion on body shape and movement

A variety of evidence demonstrates that emotion elicited by an experimental task can influence body shape and movement. A study of gait conducted by Michalak and colleagues demonstrated that while listening to sad music, healthy participants exhibited similar walking patterns as patients with major depressive disorder, providing evidence that movement qualities associated with depression can be triggered by external factors (Michalak et al., 2009). Hepach and colleagues showed that body shape can change in response to experimentally manipulated emotion by using depth sensor imaging technology to measure chest height during gait (Hepach et al., 2015). Adult chest heights while walking were found to change after inducing positive and negative emotions (Hepach et al., 2015). Subjects' chest heights were more elevated while imagining experiences of joy and pride than while imagining experiences of disappointment and guilt (Hepach et al., 2015). These effects have also been observed in children as young as 2 years old, with a greater increase in upper body posture while walking being associated with a more positive response to a toy (Hepach et al., 2015). In contrast, Oosterwijk and colleagues measured subjects' seated posture height while the subjects listed as many words as they could in about a minute and a half related to disappointment or pride (Oosterwijk et al., 2009). To measure posture height, the researchers used a hidden camera and measured the relative position of the top of a headset the subject was wearing (Oosterwijk et al., 2009). Results showed that subjects decreased their posture height more while generating negative words associated with disappointment than while generating positive words associated with pride (Oosterwijk et al., 2009). However, posture height was not found to increase significantly while listing pride-related words; in addition, horizontal movements reflecting forward or backward leaning did not significantly differ between the two conditions (Oosterwijk et al., 2009). The study included an evaluation of self-reported subjective feelings. Results from the questionnaires revealed that in addition to changes in posture height, the participants reported increased feelings of irritation and feeling worse about themselves after listing disappointment-related words than after listing words associated with pride (Oosterwijk et al., 2009).

Observational studies, particularly those evaluating posture in chronic conditions, support the results of experimental research. Emotions such as anxiety, fear, or stress have been shown to induce changes in muscle tension and co-contraction (Sainsbury and Gibson, 1954; Carpenter et al., 2001; Pluess et al., 2009; Luijcks et al., 2014; Wuehr et al., 2014), and a feeling of muscle stiffness or tightness has been subjectively described as accompanying anxiety (Sainsbury and Gibson, 1954). Depression has long been associated with physical symptoms, including slowed movement (Gupta, 2009; Buyukdura et al., 2011), slumped posture (Buyukdura et al., 2011), muscle tension (Nyboe Jacobsen et al., 2006; Gupta, 2009), and pain (Nyboe Jacobsen et al., 2006). Recent observational studies have documented a relationship between certain markers of posture, such as inclination of the head and protrusion of the shoulders, with usual sadness (Rosario et al., 2013, 2014) and a difference in posture during episodes of depression vs. periods of remission in patients with major depressive disorder (Canales et al., 2010). Canales and colleagues observed that depressed patients showed an increase in head flexion and thoracic kyphosis compared to healthy controls (Canales et al., 2010). Wilkes et al. (2017) recently reported that a cohort of depressed study participants exhibited more stooped posture than a previously assessed group of healthy controls from a previous study (Nair et al., 2015). These findings all highlight how emotions manifest in body posture.

Differences in the body associated with varying emotions can be seen not only in static posture but also in characteristics of movement, such as gait. Michalak and colleagues compared gait patterns of patients diagnosed with major depressive disorder to those of healthy, never depressed controls. The depressed subjects walked more slowly, had reduced arm swing and vertical head movements, and a more slumped posture (Michalak et al., 2009). These results are in line with those of prior studies, which noted differences not only in gait speed but also in gait pattern. Gait of depressed patients has been shown to be characterized by more of a lifting motion of the leg (Sloman et al., 1982), reduced stride length, and longer stride cycle duration compared to healthy controls (Lemke et al., 2000). Several studies evaluating gait dynamics have also shown a significant association between increased swing time variability, a marker of gait unsteadiness, and diagnosis of major depressive disorder (Hausdorff et al., 2004; Radovanović et al., 2014), depressive symptoms (Herman et al., 2005; Brandler et al., 2012), and fear of falling (Herman et al., 2005).

Taken together, this evidence suggests that transient emotional states and chronic mood disorders can lead to changes in body posture and movement. If the relationship between emotion and mood, on the one hand, and body posture and movement, on the other, is reciprocal, such that direct changes to body posture and movement can modulate emotions and mood states, then a reversal of the altered posture triggered by depression could serve as a potential therapeutic target. In the following sections, we explore existing evidence of the converse effects of changes in body posture and movement on emotion and cognition.

### Effects of posture and movement on affect and cognitive processes

#### Upright vs. slumped posture

Early work by Riskind and colleagues explored the effects of various postural configurations on outcomes such as persistence on a frustrating task and self-reported self-confidence, affect, and stress (Riskind and Gotay, 1982). Results of two experiments revealed that after holding a stooped posture for 3 min, with back hunched and head slumped forward, subjects were less persistent on a subsequent insoluble puzzle solving task (measured by number of attempts made to solve the puzzle) than those who held an upright physical posture (Riskind and Gotay, 1982). However, there were no significant differences observed in subjects' self-reported feelings (Riskind and Gotay, 1982). The relationship between posture and cognitive processes is complex and is dependent on context. For example, subsequent studies by Riskind showed that posture can have different effects on motivation depending on whether it is congruent or incongruent with the situation. If a slumped posture is adopted when it is appropriate, such as in response to failure, it can serve a protective role against feelings of helplessness and depression. In contrast, if a slumped posture is adopted when it is not appropriate, such as in response to success, it can undermine feelings of motivation (Riskind, 1984). An experimentally induced slouched posture has also been shown to undermine confidence in one's self-evaluations without necessarily influencing the nature of those self-evaluations (Briñol et al., 2009). The effects of adopting upright or slumped postures have also been shown to vary with both cultural background (Park et al., 2013) and gender (Roberts and Arefi-Afshar, 2007).

One prominent and controversial area of recent research on the effects of body posture on psychological outcomes has been the study of expansive postures or so-called “power poses” (Carney et al., 2015; Credé and Phillips, 2017; Simmons and Simonsohn, 2017). Social psychologists have investigated, under various conditions, the effects of adopting expansive postures typically associated with feelings of pride and empowerment. Typical power poses involve standing with hands on hips, sitting with feet on a desk and arms behind the head, or standing with hands firmly placed on a desk. Effects of these postures have been experimentally compared to those of slumped, contractive postures. An upright posture has been associated with self-reported ratings of increased self-esteem, arousal, and mood (Nair et al., 2015) while a contractive posture has been associated with ratings of decreased creativity and increased immediate stress (Kwon and Kim, 2015). Although adopting and holding power poses was initially shown in some cases to influence endocrine markers of power, such as increased testosterone and decreased cortisol (Carney et al., 2010), and to increase unconscious and conscious feelings of power (Carney et al., 2010; Huang et al., 2011; Ranehill et al., 2015), the influence of power-posing on testosterone and cortisol has not been upheld in replication studies (Ranehill et al., 2015), though effects of increased self-reported feelings of power have been more consistently demonstrated (Ranehill et al., 2015).

#### Emotional postures

In addition to testing the effects of expansive vs. contractive postures, several experiments have been designed to test the effects of adopting emotionally expressive postures and facial expressions, particularly those associated with fear, anger, sadness, or happiness. In some cases, subjects were given specific instructions for how to position the body or facial muscles based on expressions described or investigated previously (Duclos et al., 1989; Flack, 2006). For example, for the posture reflecting sadness, subjects were instructed to sit in a chair with hands in lap, head dropped, and upper body limp (Duclos et al., 1989; Flack, 2006). Subjects were asked to hold the posture for several seconds and subsequently rated their feelings (Duclos et al., 1989; Flack, 2006). Another method that has been used for manipulating posture is to ask subjects to imagine and simulate the expression they would have if they felt a certain emotion (Schnall and Laird, 2003).

Researchers have found that adopting these postures tended to induce the subjective experience of the associated emotion in the participants (Duclos et al., 1989; Schnall and Laird, 2003; Flack, 2006). However, the effects were stronger in subjects identified as being more responsive to personal cues, i.e., cues from their own expressions, vs. situational or environmental cues (Duclos et al., 1989; Schnall and Laird, 2003). In the study conducted by Schnall and colleagues in 2003, holding emotional postures associated with happiness, anger, and sadness not only influenced self-reported emotions of responders, but also influenced the affective valence of recalled memories on a subsequent autobiographical memory recall task (Schnall and Laird, 2003). For example, those who held an angry posture tended to recall more anger-related memories (Schnall and Laird, 2003).

#### Movement

Several groups have also gone beyond static postures to investigate the effects of different types of movement on emotional experience. Koch and colleagues examined the ways in which affect can be influenced by different types of movements and found that the quality of the movement (e.g., smooth or sharp) as well as the shape of a movement (e.g., movement toward or away from the body) could induce either relaxation and joy or increase tension and aggression (Koch, 2014). Shafir and colleagues used logistic regression analysis to confirm that pre-specified combinations of movement elements coded using Laban Movement Analysis (LMA) reliably elicited specific emotions (Shafir et al., 2015). Happiness was elicited by rising, upward and rhythmic movements, while sadness was elicited by downward, sinking movements (Shafir et al., 2015).

In a recent study conducted by Michalak and colleagues, subjects were asked to walk on a treadmill while real-time gait data were captured using infrared video cameras and reflective markers (Michalak et al., 2015). Subjects were not explicitly told how to walk, but rather were guided by visual biofeedback to achieve a walking pattern representative of either a sad or happy walk based on previously identified gait characteristics correlated with these moods (Michalak et al., 2009). Subjects were reliably able to change their gait pattern to be comparable to that of either depressed or happy individuals. After maintaining these walking patterns for about 17 min, self-reported mood state was collected using the Positive and Negative Affective Scale (PANAS). No significant differences in reported mood were found between the two feedback conditions (Michalak et al., 2015). However, memory bias on a memory recall task was found to differ significantly between groups (Michalak et al., 2015). Those who were in the happy walk condition recalled fewer negative words whereas those in the sad walk condition did not exhibit a clear bias (Michalak et al., 2015). Further, the degree of memory bias was positively correlated with the level of happiness displayed in the subject's gait (Michalak et al., 2015).

#### Facial expression

Much of the early work investigating whether external expression can influence internal experience has focused on feedback from facial expressions. Early studies of facial feedback demonstrated that deliberate configuration of facial expressions can induce distinct physiological changes similar to those of spontaneous emotional experience (Ekman et al., 1983; Levenson et al., 1990; Ekman and Davidson, 1993). Experimentally manipulated facial expressions not only induced autonomic changes, but also have been shown to generate subjective feelings of the associated emotion (Laird, 1974; Levenson et al., 1990; Mori and Mori, 2009). In contrast, the inhibition of facial expressions may also inhibit the subjective emotional experience. In a randomized double-blind placebo controlled trial, individuals with treatment-resistant depression showed improvement in their depression symptoms within 6 weeks of receiving Botox injections in the muscles of the glabellar region, which are associated with expression of negative emotion (Wollmer et al., 2012). There is some evidence that facial expressions are not only a means of expressing emotion but also are involved in processing of emotional information. For example, some studies have shown that after receiving Botox injections in the frowning muscles, women have increased difficulty processing emotional language (Havas et al., 2010) and correctly identifying emotion from pictures of the eyes (Neal and Chartrand, 2011).

#### Perception and emotional processing

Several studies have demonstrated that manipulating facial expressions, body positions, or movements can also affect evaluations of external content. Mori and colleagues randomized subjects to a furrowed brow condition, passively created by applying a bandage to the brow, or to a control group. Subjects were then shown neutral Tibetan characters preceded by pleasant, neutral, or unpleasant primes and were asked to rate the target images. Subjects with furrowed brows rated positively primed neutral objects more negatively than those in the control condition (Mori and Mori, 2010). In an older study conducted by Strack and colleagues, researchers either fabricated or inhibited a smile by instructing subjects to hold a pen either in their teeth or lips, respectively. Those in the smiling condition subsequently rated cartoons as more humorous than those in the non-smiling condition (Strack et al., 1988). Similar results have been shown in earlier studies (Bem, 1965; Laird, 1974). In a study extending beyond the face, conducted by Cacioppo and colleagues, subjects were instructed to induce isometric arm flexion or extension by pushing against the bottom or top of a table, respectively. These subjects were shown neutral Chinese characters and asked to rate them as pleasant or unpleasant. The ideographs presented during arm flexion were rated more positively than those shown during arm extension (Cacioppo et al., 1993). These effects may be mediated in part through varying activation of neural networks associated with approach motivation, as EEG data have shown that certain body postures, including leaning forward and smiling, increase activation in these brain areas (Davidson et al., 1990; Harmon-Jones et al., 2011; Price and Harmon-Jones, 2011).

Configuration of facial expression and posture has been shown not only to exert immediate effects on the present task but also to influence recall and emotional appraisal of memories. Holding a pen in the teeth to induce smiling during memory reactivation has been shown to decrease negative ratings of an emotional text read the previous day (Arminjon et al., 2015). A smile combined with an expansive, upright posture decreased the time it took subjects to recall pleasant memories when compared to a slumped posture (Riskind, 1983). Conversely, in the slumped posture, subjects recalled negative memories more quickly and remembered experiences that were more unpleasant than while in the upright posture (Riskind, 1983). Similar effects were seen in currently depressed inpatients. Subjects were instructed to sit in either an upright or slumped posture while they were shown 16 positive and 16 negative words (Michalak et al., 2014). During a subsequent free recall task, those who sat in the slumped posture remembered more negative words, while those in the upright posture showed no significant difference in number of positive and negative words remembered (Michalak et al., 2014).

## Discussion

A main goal of this paper is to introduce and explore the hypothesis that body postures in Tai Chi, Qigong, and related mind-body practices may be one biological factor contributing to improvements in psychological well-being. Although results vary and their relationship is no doubt complex, literature from various disciplines reveals compelling experimental and observational evidence supporting a bi-directional interdependence of physical postures and mood. Experimental manipulations of body shape, movement patterns, and facial gestures have been shown to be associated with short-term changes in mood and cognitive behavior. Conversely, experimental manipulations of mood have led to changes in posture and movement patterns. Variability in experimental results underscores the complexity of the relationship and its dependence on context. This complexity and context-dependence require further investigation to elucidate fully. However, observational studies characterizing correlations between clinical diagnoses (e.g., depression) and posture and movement patterns add further support for the existence of a connection. Collectively, these findings support the hypothesis that the body shapes and movement patterns trained in TCQ may be associated with the improvements in psychological well-being reported in clinical trials (Jahnke et al., 2010; Wang et al., 2010a, 2014; Chi et al., 2013; Payne and Crane-Godreau, 2013; Liu et al., 2015).

Of specific relevance to mind-body practices, some of the key features of posture and movement associated with improved mood that we identified in our literature review are principles typically emphasized in TCQ training. Some of these features, such as balanced muscular tone during static and dynamic activities (Forrest, 1997; Wolf et al., 1997; Gatts, 2008; Cho, 2014; Zorzi et al., 2015) and steadier gait dynamics (Manor et al., 2014; Wayne et al., 2015), have been objectively documented in a handful of TCQ studies. Many other features associated with improved mood, such as upright vs. slumped torso, minimized neck flexion, smooth vs. sharp movement rhythms, relaxed arm swings during weight shifting and gait, and soft facial expressions (e.g., relaxed jaw, soft eye gaze), have not been objectively measured, but are emphasized in training manuals (Lo et al., 1979; Wile, 1996; Wayne and Fuerst, 2013). However, in no studies to date that we are aware of have characteristics of TCQ postures or movement been correlated with affect or psychological well-being. Thus, it is premature to attribute any possible interpretation of specific TCQ postures or movement patterns to affect or psychologically relevant clinical outcomes. Elucidating the influence of specific static and dynamic postures on mood and related cognitive behaviors could lead to more targeted and effective mind-body training regimens for the treatment of mood disorders, psychological comorbidities commonly associated with chronic health conditions (e.g., chronic pain, heart failure, Chronic Obstructive Pulmonary Disease), and general well-being. Additionally, studies using TCQ and related mind-body exercises as an experimental tool, in concert with evaluations of the neural and psychosocial basis of the interdependence of posture and affect, could lead to fundamental advances in the broader field of embodied cognitive science.

Although existing evidence supports the plausibility of the direct role of postures in mediating the benefits of TCQ, verifying this hypothesis empirically poses significant methodological challenges. A primary obstacle is that of isolating the postures from the myriad of additional factors influencing the TCQ practitioner. During a typical training program, in addition to following verbal, modeled, and/or touch-guided choreographical instruction from a TCQ teacher (e.g., arm and leg positions, torso orientation, etc.), individuals also are commonly given instruction in breathing patterns, attentional focus, mental imagery, and philosophy (e.g., “go with the flow,” “don't try too hard”). Subtle differences in the language used during instruction may influence the quality of the students' posture and movements (Cohen et al., 2015). Sometimes training includes motivational elements in the form of encouragement or critical feedback. In addition to these instructor-student interactions, students typically learn and practice in groups, surrounded by others who serve as additional visual examples to inform their own postures and movement. These classmates often provide rich sources of psychosocial interactions immediately before and after a group training session (Fischer et al., 2014). Finally, the physical environment within which group training takes place may also influence posture, mood, and the other factors discussed above. It is easy to imagine that situating the above group-training in a repurposed hospital conference room (typical in many clinical studies), might influence multiple biologically relevant processes in quite different ways compared to groups situated within dedicated TCQ schools (surrounded by art and related symbolic icons) or in outdoor natural settings (deep in nature or a park in the middle of an urban Asian city). Collectively, these social interactions and environmental factors have the potential to create a culturally rich context for meaning, remembering, and perhaps even amplifying certain therapeutic experiences during TCQ practice. Viewed from the framework of embodied cognitive science (Varela et al., 1991) and cognitive ecology (Hutchins, 2010), posture and mood, and more broadly behavior, must be viewed as properties of the entire coupled brain-body-environment system and cannot in general be properly attributed to any one subsystem in isolation from the others (Chiel and Beer, 1997).

### Future directions

As an initial experimental approach to verifying our hypothesis, we propose relatively short-term, well-controlled, proof-of-concept studies. By manipulating individual or multiple targeted elements of posture and movement phrases, the relative contribution of different components of a larger TCQ training regimen on affect and clinical outcomes could be explored. Toward this end, tools developed in the study of dance, expressive arts therapy, and related fields of psychology may be particularly applicable to the study of TCQ within an embodied cognitive framework. For example, LMA is a well-developed method and language for describing, visualizing, interpreting, and documenting various components of human movement (e.g., shape, use of space, and effort; Bartenieff et al., 1984; Shafir et al., 2015). Other systems for classifying movements that have been employed in the context of embodied cognition include the Kestenberg Movement Profile (Amighi, 1999) and the Body Action and Coding System (Dael et al., 2012a,b). In future research, LMA or other movement classification systems could be applied to TCQ to identify specific movement features that may influence mood.

Posture is one obvious parameter that could be experimentally manipulated. For instance, the effects of specific and common TCQ stances and postures vs. “sham” postures on mood and psychophysiology could be evaluated. More specifically, mood and psychophysiology could be measured after instruction that explicitly emphasizes proper alignment and/or choreography of a specific training element, as compared to instruction lacking this emphasis or incorporating poor biomechanics (e.g., proper vs. improper neck and shoulder positions). Even more precisely, the impact of postural modifications communicated through an instructor's physical manipulation, vs. verbal instruction (including imagery), vs. non-verbal visual cues (e.g., mirroring instructor) could be used to begin to experimentally understand how psychosocial and sensory-motor processes through which TCQ training generally occurs impact posture and mood. These study designs would logically be implemented in both novice and experienced practitioners to evaluate the potential impact of long-term training. Collectively, these shorter-term experiments would inform key postural and movement features and outcomes to consider in longer-term clinical studies.

It is not yet clear whether changes in posture and movement quality can impart meaningful benefits in a clinical population and whether such effects can be sustained over time. More definitive answers regarding the role of TCQ-related posture and movement training in influencing clinical outcomes such as depression and anxiety will require longer-term controlled clinical trials. Lessons learned from the shorter-term experimental studies like those outlined above (i.e., salient postural and movement features) could be integrated into longitudinal trials with clinical populations. In such trials, participants might be exposed to a course of TCQ training (e.g., 6 months). Paralleling repeated measures (e.g., 2 month intervals) of primary clinical outcomes (e.g., mood), various dimensions of posture and quality of movement would be characterized. Methods such as regression analysis, mediation analysis, and other statistical approaches could then be used to address the hypothesis that clinical changes are at least in part attributable to postural and movement patterns. Such an approach has been used in Tai Chi studies that have shown that up to 70% of the clinical benefit of Tai Chi on pain and disability in patients with chronic low back pain is attributable to changes in cognitive constructs such as catastrophizing and rumination (Hall et al., 2016). We suggest beginning with a clinical population such as individuals diagnosed with major depressive disorder, generalized anxiety disorder, or post-traumatic stress disorder, as there is both a clinical need and significantly more room for change over shorter periods of time compared to healthy populations. Such studies would provide insight into the longer-term effects of postural training, in contrast to the largely acute effects summarized above.

### Challenges

Obtaining biometrically accurate measurements of posture also poses practical methodological and instrumentational challenges. Several different methods have been used (see do Rosário, 2014 for a review), but most have not been validated or standardized and are prone to error or associated with high costs (do Rosário, 2014). Visual observation has been used in some studies to assess posture (Lewis et al., 1992; Tracy and Matsumoto, 2008), but the subjective nature of such judgment introduces substantial bias. One of the most basic and commonly used methods of posture measurement is photogrammetry, which involves measuring the angles and distances between body landmarks in photographs of the frontal and/or sagittal plane (do Rosário, 2014). This method was applied in the observational studies correlating posture and depression (Canales et al., 2010; Rosario et al., 2013). However, protocols for the use of photographs in posture measurement have not been standardized; studies have used different sets of anatomical landmarks, setups, and processing approaches (do Rosário, 2014). There is also much room for error when using this approach, as placement of markers on anatomical points may not always be accurate or consistent (do Rosário, 2014).

Posture is dynamic rather than fixed, continuously shifting from moment to moment. Because photographs capture only a single instance of posture, they do not capture the fluctuations of posture over time. Furthermore, overtly photographing an individual's posture inhibits measurement of natural posture, as awareness of a photo being taken is likely to trigger conscious postural adjustment. Oosterwijk and colleagues addressed some of these challenges by instead using a hidden video camera, allowing for unobtrusive evaluation of posture change over time (Oosterwijk et al., 2009). Technological developments have resulted in novel methods of biomechanical assessment of posture, such as depth sensor imaging technology (Hepach et al., 2015) and 3D motion capture systems (Michalak et al., 2015), which provide improved accuracy, but with the drawback of high cost (do Rosário, 2014). Wearable sensors provide promising opportunities for naturalistic measurement of posture and movement (Wong et al., 2007); however, consensus is needed for selecting anatomical landmarks that provide the greatest accuracy (do Rosário, 2014). Although many promising methodologies exist, validation and standardization of exact protocols are lacking.

Because of the interdependent nature of posture and mood, and because both posture and mood are biologically intertwined with other therapeutic components inherent to TCQ training (e.g., breathing, imagery, heightened interoception, social interactions), evaluating the impact of posture on mood will not make substantial progress by relying on simplistic causal models. Rather, understanding how body shape and movement influence mood in the context of TCQ training will require an ecological and systems biology perspective. A systems biology approach has been advocated for investigating how multi-modal TCQ training regimens influence health (Wayne and Kaptchuk, 2008a,b; Wayne et al., 2013). A handful of studies supports the use of both non-linear dynamical metrics (e.g., Multiscale Entropy, Detrended Fluctuation Analysis) and dual-task testing paradigms (i.e., cognitive plus motor challenges) for characterizing TCQ's ability to enhance physiological “cross-talk” during standing balance (Manor et al., 2013; Wayne et al., 2014), gait (Manor et al., 2014; Wayne et al., 2015; Gow et al., 2017) and other functional tasks (Lu et al., 2013, 2016). Although this more ecological approach has highlighted TCQ's positive impact on enhancing interactions between executive attention and postural control, it has not yet specifically informed the investigation of the interdependence of posture, musculoskeletal dynamics and mood. Investigation of the intersections among multiple factors will require large sample sizes, a variety of combined approaches, and must include both quantitative and qualitative methods to address successfully the validity of the proposed role of posture in TCQ. Combined with qualitative methods and measures of changes in interoception skills, sense of social connectivity, etc., such studies would provide insight into the relative importance of each active ingredient of TCQ. Additional quantitative measures that would probe the underlying biological causal factors of any observed interdependence between posture and mood could include measurement of changes in the brain, peripheral nerves, connective tissue, and musculoskeletal system.

## Conclusion

Posture in TCQ likely contributes to TCQ's effects on mood. However, substantial further research employing mixed methods and an ecological framework based on embodied cognitive science are necessary to elucidate the nature of the relationship. Investigating this hypothesis further could begin to clarify the causal factors by which TCQ and related mind-body trainings exert their beneficial effects. An enhanced understanding of the somatic and psychological effects of TCQ presents the opportunity to enhance their therapeutic potential by enabling tailoring to specific clinical populations. TCQ and related practices may also serve as rich tools for experimentally exploring models and assumptions in embodied cognitive science, providing an avenue for augmenting our understanding of embodied cognition.

## Author contributions

KO, ET, and PW contributed to the conception of the manuscript. KO and PW wrote sections of the manuscript. ET revised critically for important intellectual content. All authors contributed to manuscript revision, read, and approved the submitted version.

### Conflict of interest statement

PW is the founder and sole owner of the Tree of Life Tai Chi Center. PW's interests were reviewed and managed by the Brigham and Women's Hospital and Partner's HealthCare in accordance with their conflict of interest policies. The others authors declare that the research was conducted in the absence of any commercial or financial relationships that could be construed as a potential conflict of interest.
